# The Evolutionary and Molecular Features of Broad Host-Range Necrotrophy in Plant Pathogenic Fungi

**DOI:** 10.3389/fpls.2020.591733

**Published:** 2020-11-12

**Authors:** Toby E. Newman, Mark C. Derbyshire

**Affiliations:** Centre for Crop and Disease Management, Curtin University, Perth, WA, Australia

**Keywords:** host generalist, broad host-range, asexuality, mating type, niche generalism, plant immunity

## Abstract

Necrotrophic fungal pathogens cause considerable disease on numerous economically important crops. Some of these pathogens are specialized to one or a few closely related plant species, whereas others are pathogenic on many unrelated hosts. The evolutionary and molecular bases of broad host-range necrotrophy in plant pathogens are not very well-defined and form an on-going area of research. In this review, we discuss what is known about broad host-range necrotrophic pathogens and compare them with their narrow host-range counterparts. We discuss the evolutionary processes associated with host generalism, and highlight common molecular features of the broad host-range necrotrophic lifestyle, such as fine-tuning of host pH, modulation of host reactive oxygen species and metabolic degradation of diverse host antimicrobials. We conclude that broad host-range necrotrophic plant pathogens have evolved a range of diverse and sometimes convergent responses to a similar selective regime governed by interactions with a highly heterogeneous host landscape.

## Introduction

Our ever-expanding global population relies on successful crop production for food. However, many factors reduce crop yield. These include biotic stress caused by bacteria, fungi, insects, oomycetes, and viruses. Crop disease caused by pathogenic fungi poses a particularly significant risk to food security. Fisher et al. (2012) estimated that if major fungal and oomycete epidemics simultaneously broke out in the five crops rice, wheat, maize, potato, and soybean, there would be a severe food shortage with enough produce left for only 39% of the global population. Understanding the pathogenesis of fungal plant pathogens is, therefore, of considerable importance.

Fungal plant pathogens exhibit distinct feeding strategies. Those that feed off living tissue are known as biotrophs, those that kill and feed off dead tissue are known as necrotrophs and those that exhibit a biphasic feeding strategy, initially colonizing as a biotrophic pathogen then switching to necrotrophy once infection is established, are known as hemibiotrophs. In this article, we refer to both hemibiotrophic and necrotrophic fungi as necrotrophs because the term “hemibiotroph” is quite poorly defined and in both instances a compatible interaction results in host cell death.

Fungi with a necrotrophic phase are far more economically damaging than biotrophs. In Australian wheat production, for example, necrotrophic leaf fungi cost the industry over twice as much as biotrophic leaf fungi at $322 million AUD per year (Murray and Brennan, 2009). Many necrotrophic fungal plant pathogens are host-specific and only cause disease on a narrow range of host species. Conversely, other necrotrophic fungal plant pathogens have a remarkably broad host-range. In fact, the host-ranges of fungi differ more than those of any other pathogen taxa (Fisher et al., 2012).

Broad host-range necrotrophic pathogens cause damage and yield loss to a wide range of economically important crops. The underlying molecular mechanisms facilitating broad host-range necrotrophy and the evolutionary driving forces giving rise to host generalism in these pathogens have not been well-defined to date.

**FIGURE 1:**
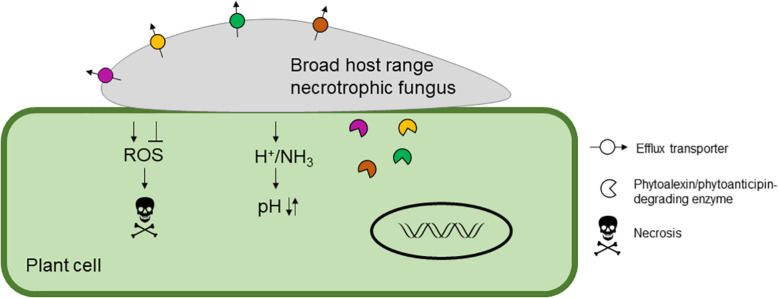
A schematic representing the molecular features of broad host range necrotrophic fungal pathogenesis. Typical molecular mechanisms to infect the host are shown including modulation of host reactive oxygen species (ROS), which is generally suppressed during initial infection and then promoted at later stages to induce necrosis. The host pH is modulated by the secretion of acid(s) to reduce the pH and/or ammonia to increase the pH resulting in suppression of host immunity and increased virulence. Finally, host-derived antifungal secondary metabolites, phytoalexins and phytoanticipins, are detoxified by efflux or degradation. Typical broad host range necrotrophic fungi harbor an array of detoxification enzymes and transporters indicated by the different colors.

In this review, we aim to discuss what is known about broad host-range necrotrophic fungi and how this differs from necrotrophic fungi with narrower host ranges. We focus on the evolutionary processes associated with the generalist lifestyle and common molecular features involved in establishment of compatible interactions with numerous host species. Table 1 summarizes the different aspects of broad host-range necrotrophy that are covered in this article in relation to 13 well-studied plant pathogens with necrotrophic stages in their lifecycles; Figure 1 specifically summarizes the molecular features of host generalism that are discussed. Host range for these species was determined using the United States Department of Agriculture Agricultural Research Service website, accessible at time of writing via https://nt.ars-grin.gov/fungaldatabases/. Only host species that could be verified in the literature were counted for pathogens that infect fewer than five plant families.

**TABLE 1:** A selection of 14 pathogen species and their host ranges with a summary of the various evolutionary and molecular features discussed in this article.

Pathogen	Asexuality	Mating type structure^†^	No. host families^††^	No. host genera	ROS modulation^†††^	pH modulation^††††^	Host antimicrobials^†††††^
*Rhizoctonia solani*	Prevalent	Het.	169	823	G	Acid.	Camalexin, cyclobrassinin, sakuranetin
*Botrytis cinerea*	Limited	Het.	146	593	G	Acid. + Alk.	Glucosinolates, camalexin, ɑ-tomatine, avenacin, avenacosides, digitonin, terpinolene, resveratrol, pterostilbene
*Alternaria alternata*	Prevalent	Het.	117	407	G	Acid. + Alk.	ɑ-tomatine
*Sclerotinia sclerotiorum*	Prevalent	Hom.	88	357	G	Acid. + Alk.	Glucosinolates, camalexin
*Fusarium graminearum*	Limited	Hom.	28	93	ND	ND	Benzoxazinoids
*Alternaria brassicicola*	Prevalent	Het.	12	28	G	Acid.	Glucosinolates, camalexin, isalexin.
*Monilinia fructicola*	Prevalent	Het.	9	19	G	Acid.	ND
*Pyrenophora tritici-repentis*	Limited	Hom.	2	21	HS	ND	ND
*Zymoseptoria tritici*	Limited	Het.	2	8	ND	ND	ND
*Pyrenophora teres*	Limited	Het.	1	19	HS	ND	ND
*Parastagonospora nodorum*	Limited	Het.	1	6	HS	ND	MBOA
*Dothistroma septosporum*	Limited	Het.	1	4	ND	ND	ND
*Ascochyta rabiei*	Prevalent	Het.	1	4	ND	ND	Medicarpin, maackiain

*^†^Het. = heterothallic; Hom. = homothallic. ^††^Number of host families and genera was derived from the United States Department of Agriculture Agricultural Research Service accessible at https://nt.ars-grin.gov/fungaldatabases/at time of writing. ^†††^HS = host-specific; G = general; ND = not documented. ^††††^Acid. = acidification; Alk. = alkalinization. ^†††††^Referring to breakdown of by pathogen.*

## The Biotic Landscape in Which Plant Pathogens Operate

### The Plant Immune System

For a long time it was thought that plant immunity functions on two levels. On one level, plants possess non-host resistance. This is based on recognition of molecular patterns broadly conserved among microbes called MAMPs (short for “microbe-associated molecular patterns”) (Ausubel, 2005). This acts as a general barrier to microbial species with pathogenic potential and it is known as MAMP-triggered immunity (MTI). A second level exists for pathogenic species that have evolved ways of bypassing MTI. Typically, these species produce molecules, loosely termed “effectors,” that suppress MTI. The effectors of these adapted pathogens may be recognized by genes in host populations called “R” (for “resistance”) genes leading to activation of a second line of defense in a process that has been termed “effector-triggered immunity” (ETI). This interaction between specific loci in plant and pathogen is known in plant pathology as the “gene-for-gene” model (Flor, 1971). To avoid this line of defense, adapted pathogens must lose or modify their effectors that correspond with the R genes present in the host population. In turn, the plant population must gain new R genes to recognize different effectors if it is to maintain resistance to the pathogen. The constant evolutionary back-and-forth between pathogen effector molecules and plant R genes results in a classic evolutionary arms race and is described as the “zigzag” model of plant parasite interactions (Jones and Dangl, 2006).

Although the general principles of this model are supported by empirical data, the real picture, as with everything in nature, is likely a lot more complex. Indeed, the two levels of the plant immune system are deeply intertwined and in many cases it may be difficult to distinguish between the molecular mechanisms underlying non-host immunity and the more specialized ETI (Schulze-Lefert and Panstruga, 2011). Furthermore, necrotrophic pathogens have evolved proteins and metabolites that elicit rather than suppress ETI in plants (Liu et al., 2017). This is because the ETI response often involves localized programmed cell death (PCD), which restricts growth of biotrophs but provides a metabolizable substrate for necrotrophs. These proteins and metabolites, often described as toxins or necrotrophic effectors, may interact with the same types of R genes that produce a resistance response to biotrophs. This results in a coevolutionary pattern that is a mirror image of the one described by the gene-for-gene model. In this instance, presence of a specific host gene that corresponds with a specific pathogen gene results in susceptibility rather than resistance. This kind of interaction has been described in various pathosystems as the matching allele or “inverse” gene-for-gene model of coevolution (Thrall et al., 2016).

A refinement of the gene-for-gene model was proposed by Cook et al. (2015) to address its many imperfections. The new model, called the “invasion model,” unites MTI and ETI in a common framework, which describes plant immunity as a general system to detect and prevent invasion of pathogenic species. It does away with the dichotomy between MTI and ETI response systems, instead suggesting that they exist on a gradient from those that detect broadly conserved pathogen molecular motifs to those that detect more taxonomically constrained ones. The molecular features of pathogens are not always strictly either MAMPs or effectors, and can have structural roles or immune system suppression or activation roles or both. This general framework is well-supported by the wealth of recent empirical data on plant pathogens. However, we find that using the terms ETI and MTI to describe particular aspects of the plant immune system, albeit ones that exist on a continuum, is convenient and will thus use aspects of both models to make points in this review.

### The Landscape of Plant Immunity in a Community of Host Species

Host generalism could theoretically develop through two macro-evolutionary processes. Either a single pathogen lineage maintains pathogenicity as its host diversifies into multiple different species, or, a specialist pathogen expands its host range. General consensus is that pathogens commonly switch between hosts and expand their ranges and long-term maintenance of pathogenicity on a diversifying plant lineage is rare (Thines, 2019). Generally, taxonomic distance between hosts is negatively correlated with the likelihood of host range expansions in pathogens. This implies that there are qualitative differences in immunity between plant families that select for quite specific, and mostly non-transferrable, molecular capabilities in pathogens (Schulze-Lefert and Panstruga, 2011).

There are several examples of such taxonomically restricted aspects of plant immunity. For instance, the Capparales is the only order of plants containing species that produce indole glucosinolates (Humphry et al., 2010). These compounds are broadly toxic to microbial species (Sotelo et al., 2015) and deployed in response to pathogen attack (Xu et al., 2016). Parasites of Capparales plants may have ways of avoiding the negative impacts of indole glucosinolates. For example, the brassicaceae-specific pathogen *Alternaria brassisicola* appears less sensitive to indole glucosinolates than other fungi (Buxdorf et al., 2013), as discussed further in section “Metabolic Flexibility is a Key Adaptation to Infection of Multiple Host Species.”

Such taxonomically restricted immunity mechanisms may increase the probability of parasite host range expansions within particular plant families. However, some species can parasitize a truly broad range of hosts from diverse and evolutionarily distant families. For example, the pathogen *Sclerotinia sclerotiorum*, which is in the Sclerotiniaceae clade, infects at least 75 different plant families (Boland and Hall, 1994). Navaud et al. (2018) showed that the common ancestor of pathogens in the Sclerotiniaceae likely infected plants in the Fabidae clade. This implies that, in *S. sclerotiorum*, host range has recently been expanded to many unrelated plants. What evolutionary and environmental processes promote the emergence of such niche generalism are an important focus of molecular ecological research. In the following sections, we summarize current understanding of this phenomenon and how it relates to necrotrophic plant pathogens. We also discuss ways in which the molecular arsenals of host generalist phytopathogens might be adapted to interaction with diverse plant immune systems.

## Why Does Host Generalism Evolve?

Niche breadth is a fundamental concept in ecology. It eludes simple definition as niches do not exist on a single axis. For example, one niche axis could be a range of temperatures and another could be a range of soil types. The different axes may be correlated and may interact in different ways. Therefore, the niche axis must be defined before investigation of the evolutionary forces affecting niche breadth is possible. In necrotrophic plant pathogens, an obvious axis of niche breadth is the range of susceptible hosts. The evolution of host generalism can thus be considered synonymous with evolution of niche breadth in a more abstract sense. For this reason, in the following paragraphs, we apply several theoretical developments on the evolution of niche breadth to the evolution of host range in necrotrophic fungal phytopathogens.

According to Whitlock (1996), competition with specialists is likely to increase the extinction rate of generalists. The fixation rate of favorable alleles, not considering those that are advantageous on one host and deleterious on another, in host generalists can be simply parameterized as 2*ps*. This expression has the parameters *p*, which describes the probability of meeting a host on which the allele is favorable, and *s*, the selection strength. In specialist species, adaptation may be faster as selection strength is not scaled to *p*. The specialist parasites spend 100% of their time on a single host, so selective pressure on favorable alleles for infecting that host is continuous. Therefore, host generalists would mostly not be favored by natural selection as they are unable to compete with specialists in any given environment. Joshi and Thompson (1995) suggest that restrictions to the fitness of generalists may also be caused by evolutionary trade-offs between advantages of alleles on one host species and their deleterious effects on another. Such negative correlations between fitness of alleles on different species may, again, increase overall selection for host specialists in populations. However, such theoretical trade-offs are seldom observed in nature and may be less important determinants of host range evolution than previously thought (Bennett and Lenski, 2007; Sexton et al., 2017).

Yet, despite these theoretical constraints, generalist pathogens exist; but why? There is some evidence that their occurrence is strongly influenced by niche heterogeneity. The environment to which a pathogen is exposed may vary temporally and spatially. Most studies agree that a large amount of variation in the environment on a time scale that is short relative to the population generation time promotes generalism (Sexton et al., 2017). Spatial variation may have a similar effect to temporal variation if the population is widely and randomly dispersed. In a spatially heterogeneous environment, organisms that do not disperse very widely will experience a similar environment across generations. Similarly, species that exhibit a behavioral preference for a particular niche are unlikely to experience differences in niche between generations. Since fungi are often both widely and randomly dispersed onto hosts, this theory would predict that plant pathogenic fungi inhabiting regions of high host species diversity would exhibit greater levels of generalism.

To our knowledge, there are no studies that have formally tested this hypothesis for plant pathogenic fungi. However, this may be the case for scale insects (Hardy et al., 2015), which also feed on plants. In the tropics, where plant species diversity is high, the proportion of polyphagous scale insect species is also high. This contrasts much of the previous literature on host preference in insects, which shows an increase in specialization in the tropics (Forister et al., 2015). The reason that scale insects are different is that they are, similarly to fungi, passively dispersed. In tropical populations where the probability of meeting multiple different hosts with each dispersal is high, the costs of specialization outweigh its benefits. Other insects that were studied hitherto are not passively dispersed as their offspring are deposited in eggs laid on preferred hosts by discerning females. For these species, the stable climate and host communities of the tropics offer a temporally consistent environment between generations. This contrasts temperate climates, where species that are not passively dispersed are more likely to experience temporal variations in host availability due to the less stable environment.

Intraspecific competition for resources may also have an impact on niche breadth. For example, Bolnick (2001) maintained large and small populations of *Drosophila melanogaster* on a heterogeneous food source with a range of cadmium content. At the outset, both populations performed poorly on high cadmium food sources. The larger population experienced greater intraspecific competition as there were more individuals competing for the low cadmium food sources. This led to a quicker expansion in the range of cadmium content tolerated over the smaller population. This confirmed several earlier predictions that this would occur (Wilson and Turelli, 1986).

Overall, we find that there are few empirical studies on fungi that test hypotheses regarding host generalism that arise from population genetic and ecological theory. The passive and geographically broad dispersal of many fungi should make them more prone to host generalism than many other taxa, especially in geographic regions where host species richness and/or intraspecific competition in pathogens are high. The contemporary broad geographic ranges of agricultural pests, which tend to be the most well-characterized species, are not relevant to the context under which they previously evolved. Therefore, for ecological research purposes, considerable effort is required to delineate the geographical origins and centers of diversity of fungi. This is becoming feasible for many pathogens as genome sequencing data are becoming cheaper. However, pathogen populations should be carefully chosen for such analyses, so that they are both sufficiently large and balanced.

## Asexuality as an Emergent Property of Host Generalism

In our review, we take the angle of Taylor et al. (2015) who suggest that true asexuality is not a likely state for a eukaryote. The first reason for this angle is based on the empirical observation that, given a comprehensive population sample, all fungi seem to have a recombining population structure. The second is that there are several evolutionary constraints on strict clonality in eukaryotes. Foremost among these is the accumulation of deleterious mutations in non-sexual lineages; a phenomenon formally described as Muller’s ratchet (Drenth et al., 2019). Put simply, beneficial and neutral mutations are able to become separated from deleterious mutations in sexual populations, whereas they are confined to one genetic background in clonal lineages.

In fungal plant pathogens, clonality can be pervasive. However, the universal existence of some degree of sexual recombination makes clonality a quantitative rather than qualitative phenomenon. In this review we distinguish between pathogens that exhibit “prevalent” asexuality and those that exhibit “limited” asexuality (Table 1). Those that exhibit prevalent asexuality include species such as *Alternaria alternata*, for which population studies typically identify multiple identical genotypes separated by great geographical or temporal distances (Meng et al., 2015). Those with limited asexuality include species such as *Zymoseptoria tritici*, which typically exhibits extremely high levels of genetic diversity and evidence of rapid linkage disequilibrium decay in natural populations (Hassine et al., 2019). We align the species *Sclerotinia sclerotiorum* with the pathogens exhibiting prevalent asexuality. Although this species is homothallic and technically obligately sexual, widespread selfing leads to a highly clonal population structure (e.g., Hambleton et al., 2002; Lehner et al., 2017). Recombination in these populations is evident from linkage disequilibrium decay analysis as in other species with significant levels of clonality (see Attanayake et al., 2014 for recombination in *S. sclerotiorum* and Meng et al., 2015 for recombination in the largely asexual species *A. alternata*). However, from a population genetics perspective, a clonal population structure is likely to be subject to the same evolutionary forces regardless of whether it is derived from frequent selfing in a homothallic species like *S. sclerotiorum* or true asexuality. We acknowledge here that the level of outcrossing that occurs in natural populations of *S. sclerotiorum* is an issue that has not been settled in the literature (Attanayake et al., 2019).

The amount of host species a pathogen can infect may have profound impacts on its mode of reproduction. In many taxa, parasitic species with broad host ranges exhibit a greater degree of asexuality than those with narrow host ranges (Gibson, 2019). Although this phenomenon has not been formally addressed in plant pathogenic fungi, among our sample of 13 plant pathogens asexuality appears more prevalent among host generalists (Table 1). This is in accordance with a similar observation in entomopathogenic fungi (Hu et al., 2014).

The potential selective forces behind this increase in asexuality among generalist parasites are reviewed by Gibson (2019), who comes to several broad conclusions. According to Normark and Johnson (2011), host generalists may have significantly larger populations than host specialists. If density per host and geographic range are equal between a specialist and a generalist inhabiting a diverse community of host species, the generalist population will be larger as it is able to infect more hosts. Sexual reproduction is very important in small populations as natural selection is less effective. There is a substantial effect of genetic drift in small populations and a lower probability of the appearance of new advantageous alleles. Therefore, selection favors a system under which advantageous mutations are separated from potentially deleterious mutations through recombination. In large populations, natural selection is much more efficient, which may reduce the need for sexual reproduction. The chances of a favorable allele arising in a given time period are quite high in a large population, and many favorable mutations may appear in non-deleterious genetic backgrounds (Ross et al., 2013).

Viewing host generalism from a slightly different angle, the infamous “Red Queen Hypothesis” (Van and Van Valen, 1973; Bell, 2019), suggests that sexual reproduction is favored when advantageous alleles become disadvantageous in subsequent generations. Specialist parasite lineages that infect the most common host genotypes would gradually degrade their resources. This would result in negative frequency-dependent selection favoring new, rare pathogen genotypes that can infect undepleted host genotypes. Sexually reproducing lineages would be selected for as they are more likely to produce novel, and at first rare, combinations of alleles.

This can be reconciled with the gene-for-gene and matching allele/inverse gene-for-gene models of plant–pathogen interactions in the following way. Pathogen lineages that are undetected by the most common R gene complement in the host population would gradually degrade this host resource and select for rare host genotypes with R genes that recognize their effector complements. Selection would in turn favor rare pathogen genotypes that have novel combinations of effectors that maximize infectivity whilst avoiding the new dominant R gene complements in the plant population. Such novel combinations would be more likely to appear in sexual lineages as they can freely acquire or lose new effectors through meiotic recombination. In pathogens that produce necrotrophic effectors, selection would initially favor lineages that infect host genotypes with the most commonly occurring combination of susceptibility genes. Again, this resource would gradually be depleted, selecting for rare host genotypes that have lost these genes. Negative frequency-dependent selection would then favor rare pathogen lineages that have novel combinations of necrotrophic effectors that correspond with the susceptibility genes present in the host population. Again, these lineages would arise more frequently from meiotic recombination, thus favoring sexual reproduction.

In both instances, selection for sexuality would be strongest when the costs of disadvantageous alleles, and thus selection strength, are high. This is clearly the case for biotrophic pathogens where a previously advantageous effector may cause complete avirulence. However, in necrotrophic pathogens, host specific necrotrophic effectors may also be under strong selection pressure. Often, only one or a few necrotrophic effectors may be the sole determinants of whether the pathogen lineage is able to infect a host lineage with a particular susceptibility gene complement (e.g., Lorang et al., 2007).

Host generalists could be under a much more “relaxed” coevolutionary regime than specialists. The Red Queen Hypothesis assumes constant antagonistic coevolution between two species to be a major promoter of sexuality. However, in widely and randomly dispersed host generalist fungal phytopathogens, antagonistic coevolution could be inconsistent. Referring again to Whitlock (1996), selection strength on generalist populations on a given host is scaled to the frequency, *p*, at which that host is encountered. If a host generalist lineage is unable to infect a particular host species, it may still survive on other host species in the environment. Thus, overall selection strength would be lower for generalists, negating the need for sexual reproduction to maintain overall fitness. Future studies could aim to elucidate the ecological reasons for asexuality among host generalist fungi, as there do not appear to be many on this topic so far.

## Broad Host-Range Pathogens Are Effective Modulators of Host Reactive Oxygen Species

The accumulation of host reactive oxygen species (ROS) is an intriguing element of plant–pathogen interactions. The involvement of ROS in plant immunity was first described nearly four decades ago in potato (*S. tuberosum*) resistance to the causal agent of potato late blight, the oomycete pathogen *Phytophthora infestans* (Doke, 1983). ROS production is now well established as an essential component of the plant immune system. Upon initial recognition of conserved microbial features (MAMPs), plant cells induce a transient burst of ROS in the apoplast within minutes (Jwa and Hwang, 2017). ROS are directly cytotoxic to invading pathogens and also act as signaling molecules to activate MTI; this is sufficient to prevent the spread of most non-adapted pathogens (Torres, 2010). However, for pathogens that have evolved to suppress MTI through secretion of immunity-dampening effectors, a second wave of ROS may await. Perception of pathogen-derived effectors by R proteins triggers ETI, which results in a prolonged, amplified accumulation of ROS and often culminates in a form of PCD termed the hypersensitive response (HR) (Jones and Dangl, 2006). This response is highly effective at impeding the growth of pathogens in a biotrophic phase. ETI-associated ROS production occurs in the apoplast as well as in organelles, particularly mitochondria and chloroplasts (Van Breusegem et al., 2008).

Paradoxically, ROS production may also be detrimental to the host. Despite the well-described role in disease resistance, the accumulation of ROS may be favorable for the pathogen. The outcome of host ROS production is highly dependent on the lifestyle of the pathogen. As ROS production is associated with the induction of PCD, high levels of ROS in the host and subsequent cell death impedes the spread of biotrophic pathogens but can aid the spread of necrotrophic pathogens, which feed off the dead tissue (Govrin and Levine, 2000). For this reason, necrotrophic pathogens have evolved various mechanisms by which to hijack the host and elevate ROS levels for their own benefit. The ability of a pathogen to modulate host ROS levels constitutes a critical mechanism by which to successfully colonize host tissues. Pathogens that have the ability to modulate ROS levels effectively can overcome host resistance and enhance virulence in multiple host species resulting in a compatible interaction and disease progression. Accordingly, effective modulation of host ROS is commonly exhibited by broad host range necrotrophic fungal pathogens.

Pathogenesis mechanisms in the archetypal broad host range necrotrophic fungal pathogen *S. sclerotiorum* are relatively well characterized and evidence suggests that it may be an adept modulator of host ROS. This is largely through the activity of the multifunctional organic compound oxalic acid (OA), which was surprisingly shown to be both a suppressor and promoter of host ROS, and a key virulence determinant on a wide range of dicotyledonous host species. According to the oxalate-dependent theory, OA functions to modulate ROS independent of its role in acidification described below (Williams et al., 2011). Secretion of OA was proposed to enable *S. sclerotiorum* to dampen ROS accumulation, thereby abrogating host defense responses during initial colonization and within a brief biotrophic phase. Once infection had been established, *S. sclerotiorum*-derived OA was shown to have the opposite effect and induce the generation of host ROS leading to spreading ROS-mediated necrosis (Cessna et al., 2000; Williams et al., 2011). This manipulation of host ROS is independent of any specific host components and, therefore, may function across a broad range of host species. It must be noted that this theory has recently been challenged by Xu et al. (2015) using targeted OA-deficient mutants. The authors concluded that low pH, not specifically OA, is required for *S. sclerotiorum* pathogenicity. A critical evaluation of the role of OA in *S. sclerotiorum* virulence is reviewed in detail by Xu et al. (2018). Interestingly, monocotyledonous plants harbor oxalate oxidases that break down OA, rendering it non-functional (Chiriboga, 1966; Lane et al., 1993). This may play a role in restricting the host range of *S. sclerotiorum* almost exclusively to dicotyledonous plant species. Indeed, transgenic oilseed rape expressing a barley or wheat oxalate oxidase gene and transgenic soybean expressing a wheat oxalate oxidase gene exhibit enhanced resistance to *S. sclerotiorum* (Thompson et al., 1995; Dong et al., 2008). Furthermore, a homolog of a gene involved in biogenesis of OA, oxaloacetate hydrolase (*OAH*), was highly induced in *Rhizoctonia solani* during infection of wheat (Foley et al., 2016). We can assume that OA would be broken down by wheat oxalate oxidase; however, *R. solani* may utilize OA effectively for infection of its dicotyledonous host species, although this remains to be confirmed.

*Rhizoctonia solani* may employ an alternative mechanism of ROS modulation to facilitate infection. Expression of the rice mitochondrial respiratory chain enzyme NADH:ubiquinone oxidoreductase (*NUOR*) was significantly upregulated during infection of a susceptible rice cultivar, whereas the *NUOR* gene of a partially resistant cultivar was significantly downregulated during infection. *NUOR* silencing in tomato and rice resulted in reduced ROS accumulation around the site of infection and enhanced resistance (Kant et al., 2019). It was proposed that *R. solani* may actively upregulate the expression of *NUOR* in order to elevate ROS accumulation, and thus induce PCD to benefit its necrotrophic lifestyle. *NUOR* is conserved in a wide range of plant species due to its fundamental role in respiration (Subrahmanian et al., 2016). In theory, this would allow *R. solani* to manipulate host ROS on a broad host range via this mechanism. *S. sclerotiorum* has also been demonstrated to target a component of the host mitochondrial respiratory chain, the cytochrome-b-c_1_ complex, using the proteinaceous secreted effector SsSSVP1, leading to host cell death (Lyu et al., 2016). Although not explicitly shown, the resulting cell death is presumably due to release of large amounts of ROS from mitochondria. The conservation of the cytochrome-b-c_1_ complex in almost all plant species suggests that SsSSVP1 may play a role in the necrotizing ability of *S. sclerotiorum* on a broad range of host species. This mechanism is in contrast to the gene-for-gene interaction demonstrated for many narrow host range pathogens, which rely on specific host targets for favorable ROS accumulation. As discussed in section “The Plant Immune System,” when necrotrophic effectors interact with R genes to elicit an HR, this results in susceptibility and is commonly referred to as an inverse gene-for-gene interaction (Fenton et al., 2009). This type of host-specific interaction has been demonstrated for the narrow host range pathogens *Parastagonospora nodorum* and *Pyrenophora tritici-repentis*, which depend on necrotrophic effectors to induce ROS production and subsequent host cell death on specific genotypes of wheat carrying cognate receptors. For example, *P. nodorum* and *P. tritici-repentis* both produce the host-specific effector ToxA, which triggers ROS production and necrosis on wheat lines carrying the R-like gene *Tsn1* (Faris et al., 1996; Liu et al., 2006). These inverse gene-for-gene interactions are critical for the necrotrophic lifestyle of host-specific *P. nodorum* and *P. tritici-repentis*, but have not been reported for broad host range pathogens.

It is becoming evident that most, if not all, necrotrophic pathogens initially colonize host tissue in a biotrophic-like manner during which ROS production can suppress or halt infection. Therefore, strategies to suppress or detoxify host ROS production provide a benefit to all pathogens when establishing infection. The transcription factor YAP1 was first identified in the yeast species *Saccharomyces cerevisiae*. YAP1 regulates the expression of antioxidant genes and is essential for tolerating oxidative stress in yeast (Schnell et al., 1992). YAP1 homologs have been identified in filamentous fungi including plant pathogens (Rodrigues-Pousada et al., 2019). YAP1 is required for hydrogen peroxide (H_2_O_2_) tolerance in all characterized YAP1-harboring fungal phytopathogens but essential for virulence only in some. Of the pathogens in Table 1, YAP1 is required for virulence of broad host range pathogens *Alternaria alternata*, *A. brassicicola*, and *Monilinia fructicola* but not for the broad host range pathogen *B. cinerea* or the narrow host range pathogens *Fusarium graminearum* and *Z. tritici* (Mendoza-Martínez et al., 2020). It is possible that *F. graminearum*, *Z. tritici*, and *B. cinerea* utilize alternative signaling pathways to cope with potentially damaging host ROS. Indeed, *B. cinerea* depends on the transcription factor BcLTF1 for oxidative stress tolerance and full virulence. When inoculated on *Phaseolus vulgaris* leaves, a *B. cinerea bcltf1* deletion mutant was unable to suppress host ROS accumulation, resulting in increased H_2_O_2_ accumulation and slower spread of the lesion when compared to the wild-type. This is due to the altered expression of ROS-related genes that are controlled by BcLTF1 (Schumacher et al., 2014). The mutant phenotype is reminiscent of an OA-deficient *S. sclerotiorum* mutant albeit with a weaker effect on virulence.

In cases where narrow host range pathogens encounter a resistant host genotype, they are often impeded in the asymptomatic, biotrophic-like phase by a host defense response that includes ROS production. For example, *Z. tritici* infection of a resistant wheat cultivar resulted in significantly higher amounts of H_2_O_2_ when compared to infection of a susceptible cultivar, which correlated with inhibition of pathogen growth (Shetty et al., 2003). Similar findings have been shown in resistance of lentil genotypes to *Ascochyta lentis* (Sambasivam et al., 2017). Presumably, these pathogens lack the ability to suppress the damaging host ROS production on some host genotypes.

## Fine-Tuning of Host pH Is Widespread Among Broad Host-Range Necrotrophs

Pathogenic fungi have evolved mechanisms to modify the ambient pH of host tissue to facilitate infection. The well-conserved Pal/Rim alkaline signaling pathway in fungi senses and responds to environmental pH (Selvig and Alspaugh, 2011). This allows pathogens to fine-tune their environment to a favorable pH through acidification or alkalinization. The benefit of pH modulation is due to its effect both on host resistance and pathogen virulence. Firstly, host pH impacts the ability of the host to mount an effective defense response. The well-characterized MTI-associated ROS burst is inhibited at a low pH (Legendre et al., 1993). In many plant species, induction of a ROS burst is associated with an increase in pH. In fact, in French bean, merely exposing cells to an alkaline environment triggered ROS production (Bolwell et al., 1995). Therefore, rapid acidification of host tissue by fungal pathogens functions to suppress MTI, thereby facilitating initial colonization in the host.

Interestingly, analysis of plant pathogenic species in the Sclerotiniaceae family revealed a remarkable difference in the *in planta* expression of oxaloacetate acetylhydrolase (*oah*), which is required for OA biosynthesis, between broad host and narrow host range pathogens. Within four hours of plant infection, *oah* expression was between ten and 300 times greater in the broad host range necrotrophic pathogens *B. cinerea* and *S. sclerotiorum* compared to their narrow host range counterparts (Andrew et al., 2012). Early production of significant amounts of OA could conceivably very rapidly lower the ambient pH and suppress a PTI-induced ROS burst. The proficiency of *S. sclerotiorum* to acidify host tissue has long been known; an early study demonstrated a reduction in the pH of sunflower stems to as low as four during *S. sclerotiorum* infection (Marciano et al., 1983). Other broad host range pathogens are capable of acidifying host tissue via production of various organic acids including phenylacetic acid (PAA) and 3-methylthioproprionic acid (MTPA), produced by *R. solani*, and tenuazonic acid, produced by *A. alternata* (Meronuck et al., 1972; Bartz et al., 2012; Kankam et al., 2016).

In addition to its role in suppression of host immune responses, acidification of the host environment may also enhance fungal virulence. For example, lowering of pH influences expression of polygalacturonase (PG) cell wall degrading enzyme (CWDE)-encoding genes during *S. sclerotiorum* infection of carrot where a pH of four to five induced the highest expression (Cotton et al., 2003). The optimum pH for activity of polygalacturonases PGs also lies within this range (Favaron et al., 2004). Therefore, acidification of host tissue results in an increase in both expression and activity of CWDEs thereby facilitating penetration and tissue maceration.

Similarly, acidification induces expression of a *B. cinerea* PG and is required for transcription and post-translational modification of the *B. cinerea* protease, BcACP1 (Wubben et al., 2000; Rolland et al., 2009). *B. cinerea* produces multiple acids during infection including oxalic, citric and succinic acids (Gentile, 1954; Verhoeff et al., 1988; Dalmais et al., 2011). A set of mutants in the VELVET complex are impaired in citric acid production and virulence. Virulence of the mutants could be partially restored by artificial acidification at infection sites demonstrating the requirement for acidification of host tissue (Müller et al., 2018). Similar results were found using *oah* mutants of *S. sclerotiorum*; acidification of host tissue could enhance virulence (Xu et al., 2015). It is no surprise, then, that the endogenous ability to acidify often correlates highly with virulence of broad host range necrotrophic fungi (Nagarajkumar et al., 2005; Bartz et al., 2012). As well as the aforementioned effects on fungal virulence, some acids secreted by fungal pathogens exhibit direct phytotoxicity, a function that benefits fungi with a necrotrophic lifestyle. For example, botcinic acid, MTPA, OA, and tenuazonic acid produced by *B. cinerea*, *R. solani*, *S. sclerotiorum*, and *A. alternata* respectively, all have phytotoxic activity (Visconti et al., 1987; Cutler et al., 1996; Kim et al., 2008; Kankam et al., 2016).

Despite the described benefits of acidification, some necrotrophic pathogens also benefit from prolonged alkalinization of the host environment. Alkalinization has been reported for the broad host range pathogen *A. alternata*, several *Colletotrichum* species and *F. oxysporum* (Fernandes et al., 2017). In all cases, the increase in pH is driven by secretion of ammonia. *A. alternata* increases pH via ammonia secretion during infection of fruits; this has been demonstrated in persimmon, tomato and melon. Ammonia production and pH increases varied between fruits, suggesting that *A. alternata* can sensitively perceive the ambient pH prior to fine-tuning. In a manner similar to the acidic induction of PG expression in *S. sclerotiorum*, an increase in pH induced expression of an endoglucanase and enhanced the spread of disease (Eshel et al., 2002).

During infection of sunflower cotyledons, *B. cinerea* and *S. sclerotiorum* were demonstrated to initially reduce the pH through secretion of citric and succinic acids then as the infection advanced both pathogens increased the pH through secretion of ammonia (Billon-Grand et al., 2012). This demonstrates effective fine-tuning of host pH to facilitate infection by broad host range pathogens.

Fine-tuning of host pH has a multitude of benefits for pathogenic success and perhaps the capacity to do so contributes to an expansion of host range. Indeed, robust evidence of host pH modulation appears to be rare in narrow host range necrotrophic fungi. Of the narrow host range pathogens in Table 1, none have been reported to modulate host pH during infection.

## Metabolic Flexibility Is a Key Adaptation to Infection of Multiple Host Species

An important defense response employed by plant species is production of antimicrobial secondary metabolites termed phytoalexins. Additionally, plants produce preformed antifungal agents, which are known as phytoanticipins (VanEtten et al., 1994). These metabolites function in conjunction with other defense strategies such as MTI and ETI to fend off invading pathogens. A well-studied phytoalexin is camalexin, named after the species from which it was first isolated, camelina (*Camelina sativa*) (Browne et al., 1991). Camalexin is produced by members of the Brassicaceae family and functions in resistance to multiple necrotrophic fungal pathogens including *A. brassicicola* and *B. cinerea*. It has also been shown to contribute to resistance to some hemibiotrophic and biotrophic pathogens, and the Brassicaceae-specific aphid, *Brevicoryne brassicae* (Ahuja et al., 2012). Camalexin has been shown to exert its toxicity by disruption of membranes (Rogers et al., 1996). Other phytoalexins are known to induce cytoplasmic granulation and inhibit fungal enzymes, all of which can effectively kill invading fungal pathogens and prevent the spread of disease (Arruda et al., 2016).

A study in *Arabidopsis thaliana* demonstrated a role for phytoalexins in non-host resistance to the necrotrophic fungus *Plectosphaerella cucumerina*. Tryptophan-derived metabolites were shown to be required for resistance to non-adapted *P. cucumerina* isolates. Intriguingly, mutant *A. thaliana* lines impaired in accumulation of these metabolites became hosts for the non-adapted isolates (Sanchez-Vallet et al., 2010). This indicates that phytoalexins play a role in defining the host range of necrotrophic fungal pathogens. Plant families are known to produce many different types of phytoalexins such as camalexin in the Brassicaceae, medicarpin in the Fabaceae, kauralexin in the Poaceae, capsidiol in the Solanaceae and resveratrol in the Vitaceae (Ahuja et al., 2012). To cause disease on a particular plant species, a fungal pathogen must be able to tolerate or metabolize the phytoalexins produced by the host. Therefore, an increase in host range would require an expanded pathogenic toolkit equipped to metabolize the antifungal secondary metabolites produced by the new host species. Broad host range pathogens such as *R. solani*, which has been reported on 169 different plant families, are capable of tolerating or metabolizing a wide range of phytoalexins.

One way in which plant pathogens can tolerate phytoalexins is by efflux. Over 20 years ago, an ATP-binding cassette (ABC) transporter was first identified as a pathogenicity determinant in the narrow host range rice blast pathogen, *Magnaporthe oryzae.* The gene was demonstrated to be upregulated in response to the rice phytoalexin sakuranetin, strongly suggesting a role of the transporter in efflux of antifungal secondary metabolites, which was necessary for infection (Urban et al., 1999).

A *B. cinerea* ABC transporter (*BcatrB*) was shown to provide tolerance to the Vitaceae-specific phytoalexin resveratrol and contribute to virulence on grapevine leaves (Schoonbeek et al., 2001). *BcatrB* expression was later found to be induced by camalexin and confirmed to play a role in tolerance to camalexin and virulence on *A. thaliana*, demonstrating that a single transporter facilitates *B. cinerea* infection of at least two plant families (Stefanato et al., 2009).

Another *B. cinerea* efflux pump belonging to the major facilitator superfamily (MFS) family (*mfs*G) provides tolerance to glucosinolates, a group of Brassicaceae-derived secondary metabolites. Interestingly, *mfs*G is differentially expressed in response to the presence of different glucosinolate constituents, isothiocyanates (ITCs), suggesting that *B. cinerea* can regulate expression of transporters in response to specific host compounds (Vela-Corcía et al., 2019).

The closely related generalist pathogen *S. sclerotiorum* also exhibits metabolic flexibility. A transcriptome analysis on infected tissue of two different host species of *S. sclerotiorum*, canola (*Brassica napus*) and lupin (*Lupinus angustifolius*), revealed differential expression of genes associated with detoxification and efflux of host-derived secondary metabolites (Allan et al., 2019). Further research is required to understand the extent of differential regulation of metabolic enzymes by broad host range pathogens. However, analysis of the number of MFS transporters in *B. cinerea* and *S. sclerotiorum* revealed a large repertoire harbored by both broad host range pathogens (Vela-Corcía et al., 2019). MFS transporters are involved in the transport of many substrates and we speculate that the large repertoires include some MFS transporters involved in the efflux of antifungal metabolites produced by the diverse plant species infected by these pathogens.

Plant pathogens also detoxify host-derived secondary metabolites by degradation or biotransformation. *R. solani* can degrade or biotransform camalexin, cyclobrassinin and sakuranetin, thereby facilitating infection on dicotyledonous crucifers and the monocotyledonous rice (Pedras and Okanga, 1999; Pedras and Khan, 2000; Katsumata et al., 2018). *A. brassicicola* is particularly successful on plants in the Brassicaceae family. This can at least in part be explained by its ability to detoxify many cruciferous metabolites including brassicanate A, brassinin, camalexin, cyclobrassinin, isalexin, isothiocyanates, and rutalexin (Pedras and Okanga, 1999; Sellam et al., 2006; Pedras et al., 2013; Pedras and Abdoli, 2017). *B. cinerea* has been demonstrated to detoxify an array of phytoanticipins and phytoalexins such as α-tomatine, avenacin, avenocosides, digitonin, camalexin, brassinin, cyclobrassinin, brassilexin, terpinolene, resveratrol, and pterostilbene (Sbaghi et al., 1996; Quidde et al., 1998; Afgan et al., 2002; Quidde et al., 2004; Pedras et al., 2011). The importance of phytoalexin detoxification for pathogenicity has been shown using natural isolates unable to detoxify specific phytoalexins. For example, *B. cinerea* isolates that cannot degrade the phytoalexins resveratrol and pterostilbene are non-pathogenic on grapevines (Sbaghi et al., 1996). Moreover, one *B. cinerea* strain was identified that was deficient in the α-tomatine-degrading enzyme, tomatinase, and was unable to induce lesions on tomato leaves but displayed comparable pathogenicity to tomatinase-producing strains on bean leaves (Quidde et al., 1998). Broad host range pathogens require an arsenal of detoxification enzymes to overcome the array of antifungal compounds that they encounter.

Narrow host range pathogens also employ detoxification mechanisms to facilitate compatible interactions with their respective host species. However, the phytoalexins degraded tend to be host-specific and the narrow host range pathogens lack the repertoire of detoxifying enzymes to metabolize a range of antifungal secondary metabolites (Table 1). For example, the causal agent of ascochyta blight on chickpea, *Ascochyta rabiei*, metabolizes the pterocarpan phytoalexins medicarpin and maackiain, known to accumulate in chickpea during *A. rabiei* infection (Weigand et al., 1986; Tenhaken et al., 1991). The wheat pathogen, *P. nodorum*, can degrade low amounts of the cereal phytoalexin 6−methoxy−2−benzoxazolinone (MBOA); however, MBOA becomes toxic at high concentrations (Du Fall and Solomon, 2013).

To summarize, detoxification of antifungal host metabolites is required for successful infection in many pathogens and host generalist necrotrophic fungi can detoxify a particularly wide range of host-derived secondary metabolites to facilitate their lifestyles.

## Conclusion

Broad host-range necrotrophic fungi are capable of causing disease and yield loss on many economically important crops. However, the molecular and evolutionary characteristics of these species are, thus far, often described poorly relatively to those of host specialists. Here, we have discussed common evolutionary and molecular features that set host generalists apart from specialists. Based on the literature discussed, we have begun to build a picture of a typical broad host-range necrotrophic fungal pathogen. This is a pathogen that likely evolved in a region of high host species diversity and that reproduces predominantly asexually. It has developed a highly efficient molecular toolkit, which enables it to establish disease on diverse plant species. This toolkit comprises mechanisms to modulate host ROS and pH, and detoxify a wide range of host-derived antifungal secondary metabolites. Further research is required to clarify the evolutionary bases of host generalism and to better understand the mechanisms by which they cause economically damaging disease to so many crop species.

## Author Contributions

TN wrote sections “Introduction,” “Broad Host-Range Pathogens are Effective Modulators of Host Reactive Oxygen Species,” “Fine-Tuning of Host pH is Widespread Among Broad Host-Range Necrotrophs,” and “Metabolic Flexibility is a Key Adaptation to Infection of Multiple Host Species” and helped in planning the overall structure. MD conceived of the review and overall structure and wrote sections “The Biotic Landscape in Which Plant Pathogens Operate,” “Why Does Host Generalism Evolve?” and “Asexuality as an Emergent Property of Host Generalism.” Both MD and TN proof-read the final manuscript version before submission. All authors contributed to the article and approved the submitted version.

## Conflict of Interest

The authors declare that the research was conducted in the absence of any commercial or financial relationships that could be construed as a potential conflict of interest.
